# Dimensionality Reduction of Hyperspectral Images Based on Improved Spatial–Spectral Weight Manifold Embedding

**DOI:** 10.3390/s20164413

**Published:** 2020-08-07

**Authors:** Hong Liu, Kewen Xia, Tiejun Li, Jie Ma, Eunice Owoola

**Affiliations:** 1 School of Electronics and Information Engineering, Hebei University of Technology, Tianjin 300401, China; 201821902006@stu.hebut.edu.cn (H.L.); jma@hebut.edu.cn (J.M.); bunmso@gmail.com (E.O.); 2 School of Mechanical Engineering, Hebei University of Technology, Tianjin 300401, China; 1993076@hebut.edu.cn

**Keywords:** curse of dimensionality, spatial–spectral weight manifold embedding, ground-truth classification accuracy, dimensionality reduction

## Abstract

Due to the spectral complexity and high dimensionality of hyperspectral images (HSIs), the processing of HSIs is susceptible to the curse of dimensionality. In addition, the classification results of ground truth are not ideal. To overcome the problem of the curse of dimensionality and improve classification accuracy, an improved spatial–spectral weight manifold embedding (ISS-WME) algorithm, which is based on hyperspectral data with their own manifold structure and local neighbors, is proposed in this study. The manifold structure was constructed using the structural weight matrix and the distance weight matrix. The structural weight matrix was composed of within-class and between-class coefficient representation matrices. These matrices were obtained by using the collaborative representation method. Furthermore, the distance weight matrix integrated the spatial and spectral information of HSIs. The ISS-WME algorithm describes the whole structure of the data by the weight matrix constructed by combining the within-class and between-class matrices and the spatial–spectral information of HSIs, and the nearest neighbor samples of the data are retained without changing when embedding to the low-dimensional space. To verify the classification effect of the ISS-WME algorithm, three classical data sets, namely Indian Pines, Pavia University, and Salinas scene, were subjected to experiments for this paper. Six methods of dimensionality reduction (DR) were used for comparison experiments using different classifiers such as *k*-nearest neighbor (KNN) and support vector machine (SVM). The experimental results show that the ISS-WME algorithm can represent the HSI structure better than other methods, and effectively improves the classification accuracy of HSIs.

## 1. Introduction

With the development of science and technology, hyperspectral images (HSIs) have become the main research direction in the field of modern remote sensing technology. HSIs have a large number of spectral bands, which provide detailed spectral information about objects [1,2]. However, due to the strong correlation between adjacent spectra, there is much redundant information in HSIs, which take up a large storage space and require much computation time. Moreover, when classifying HSIs, classification accuracy is subject to the curse of dimensionality [3]. In order to improve classification accuracy, a dimensionality reduction (DR) method is a necessary and feasible preprocessing measure for HSI [4,5].

A DR method aims at extracting important features of images, mapping high-dimensional data to low-dimensional space, and using the data in low-dimensional space to describe high-dimensional features [5]. In recent years, scholars have put forward many DR methods, which can be divided into the following two categories: linear dimensionality reduction (LDR) algorithms and manifold dimensionality reduction (MDR) algorithms [6]. The former includes principal component analysis (PCA) [7], linear discriminant analysis (LDA) [8], and independent component analysis (ICA) [9], and so on. These methods project images to the low-dimensional space by linear transformation and find the optimal transformation projection. However, because ground-truth features reflected in HSI are often nonlinear topological structures, important features of the images are lost if only an LDR method is used. Therefore, MDR algorithms are gradually appearing, including local linear embedding (LLE) [10], local preserving project (LPP) [11], Laplacian eigenmaps (LE) [12], and so on. By learning the intrinsic geometric structure of data, manifold learning [13] can obtain the potential manifold structure of the high-dimensional data to achieve the goal of dimensionality reduction.

The purpose of the MDR method in HSI is to find the manifold structure in the high-dimensional space. LLE [14] obtains the reconstruction weight by characterizing the local adjacency sample of the data and keeps the neighborhood relationship in the local range unchanged when mapping to the low-dimensional space. However, an LLE algorithm only determines the neighbor relationship between points and cannot describe the structural features of data. Therefore, the linear neighbor representation weight matrix of different samples is different. When the LLE algorithm is used for different samples, the algorithm needs to be re-run, which is time consuming. It has a considerably low efficiency. Wu et al. [15] proposed an improved weighted local linear embedding (WLE-LLE) algorithm, which constructs the weight matrix by calculating the Euclidean distance and geodesic distance between samples. In addition, it merges LLE with LE algorithms to form a new objective function to effectively represent the topology structure of the data. Huang et al. [16] proposed a sparse discriminant manifold embedding (SDME) algorithm, which forms a dimensionality reduction framework based on graph embedding and sparse representation methods to make full use of the prior label information. Xu et al. [17] proposed a superpixel-based spatial–spectral dimension reduction (SSDR) algorithm by integrating the similarity between space and spectrum. The mapping matrix of the spatial domain is found by using superpixel segmentation to explore spatial similarity. Pixels from the same label construct a label-guided graph to explore the spectral similarity. Furthermore, integrating the labels and spatial information contributes to learning a discriminant projection matrix. Wu et al. [18] proposed a correlation coefficient-based supervised locally linear embedding (SC^2^SLLE) algorithm, which introduces the Spearman correlation coefficient to determine the appropriate nearest neighbor points, and increases the discriminability of embedding data on the basis of supervising the LLE method. Zhang et al. [19] proposed a SLIC (Sample Linear Iterative Clustering) superpixel based for Schroedinger eigenmaps (SSSE) algorithm, which uses SLIC segmentation to obtain spatial information for superpixels of different scales and sizes. The use of an SE method yields low-dimensional data. Hong et al. [20] proposed a robust local manifold representation (RLMR) algorithm based on LLE, to learn a novel manifold representation methodology, and then combine the new method with spatial–spectral information to improve the robustness of the algorithm.

In this paper, an improved spatial–spectral weight manifold embedding (ISS-WME) algorithm is proposed to combine spatial–spectral information and manifold structure to extract the features of HSI. First, the spatial–spectral information of HSI is extracted with the Gaussian variant function. The product of the spatial distance matrix and the spectral distance matrix is then used to be the distance weight matrix. Then, the collaborative representation method is used to express the characteristics of the HSI structure. Samples from the same class are as much as possible in the same hyperplane after projection, and samples from the different classes are as far apart as possible. The structural weight matrix is obtained by combining the within-class and between-class weight representation matrices. The product of the distance weight matrix and the structure weight matrix is used as the new weight matrix. When the data is mapped from high-dimensional manifold space to low-dimensional space, it is easy to make abnormal points appear if only considering the structural distribution between the data points. Furthermore, it is easy to cause the problem of sparseness if only keeping the data nearest neighbor relationship unchanged during projection transformation. To overcome abnormal points and the sparseness problem, both the structure and neighbor sample relationship are taken into account in this paper. Finally, the model can be efficiently solved by solving the minimum eigenvalue to the generalized eigenvalue problem and obtaining a projection matrix. The main contributions of the proposed algorithm are as follows:

1. A new weight matrix is constructed to describe the structure between samples, in which the product of the spatial–spectral distance weight matrix and the structure weight matrix is taken as a new data weight matrix. Compared with the previous weight matrix, which only considers spectral distance or spatial distance, the new weight matrix integrates the spatial–spectral information and structural characteristic of the data.
2. The model not only makes the manifold structure invariant, but also preserves the nearest neighbor relationship of the samples, when the high-dimensional data are projecting to the low-dimensional space.

This paper is arranged as follows. Section 2 briefly summarizes the LLE and LE methods and reviews the related works of these models. Section 3 provides the detailed description and the solving process of ISS-WME. Section 4 compares the performance of the proposed method and other DR methods with respect to three public data sets. Finally, the conclusions and perspectives are provided in Section 5.

## 2. Related Works

### 2.1. Local Linear Embedding

Given the data set $X=\left[x_{1},\cdots {,x}_{N}\right]\in R^{D\times N}$, where $x_{i}\in R^{D}$, this denotes the ith sample with *D*-dimension features and *N* is the number of the samples. We assume that the *D*-dimensional-sample $x_{i}$ projects to d-dimension space *M*, d≪D. Therefore, the low-dimensional coordinate of the transformed data is $Y=\left[y_{1},\cdots {,y}_{N}\right]\in R^{d\times N}$, where $y_{i}\in R^{d}$. The core of the LLE algorithm is to retain $x_{i}$ and its local neighbor samples are unchanged after DR. We consider the point and its local neighbor points as belonging to the same class. Under the principle of minimizing reconstruction errors, the sample $x_{i}$ can be linearly represented by these neighbor samples. By reconstructing the weight matrix, the original space is connected with the low-dimensional embedding space. Moreover, the reconstruction weight matrix between each sample and its nearest neighbor samples is kept unchanged, and the embedding result in the low-dimensional space is obtained by minimizing the reconstruction errors. Therefore, the weight coefficient matrix of the relationship between $x_{i}$ and its local neighbors can be obtained by solving the following optimization problem [21]:

(1) $$\left\{ \begin{array}{l} \min\sum_{i=1}^{N}{\left\|x_{i}-\sum_{j=1}^{k}w_{\mathrm{ij}}x_{j}\right\|}^{2}\\ s.t.\sum_{i=1}^{k}w_{\mathrm{ij}}=1 \end{array}\right.$$

In Equation (1), $x_{j}\left(j=1,\cdots ,k\right)$ is one of the *k* samples, which is closest to X_i_(i = 1,⋯N), and $w_{\mathrm{ij}}$, andstands for the weight neighbor relationship between samples $x_{i}$ and $x_{j}$; if they are not neighbors then $w_{\mathrm{ij}}=0$. Assuming the projection of *D*-dimensional samples into *d*-dimension space, it is desirable to maintain the same linear relationship:

(2) $$\left\{ \begin{array}{l} \min\sum_{i=1}^{N}{\left\|y_{i}-\sum_{j=1}^{k}w_{\mathrm{ij}}y_{j}\right\|}^{2}\\ s.t\frac{1}{N}\sum_{i=1}^{N}y_{i}y_{i}^{T}=I,\sum_{i=1}^{N}y_{i}=0 \end{array}\right.$$

where *I* is the identity matrix and $y_{i}{=\mathrm{YI}}_{i}$. Then, we have the following: $M={\left(I-W\right)}^{T}\left(I-W\right)$, hence, Equation (2) can be changed into the following problem:

(3) $$\underset{Y}{\mathrm{argmin}}\sum_{i=1}^{N}{\left\|{\mathrm{YI}}_{i}{-\mathrm{Yw}}_{i}\right\|}^{2}=\underset{Y}{\mathrm{argmin}}\mathrm{tr}\left({\mathrm{YMY}}^{T}\right)$$

Using the method of Lagrangian multiplier, Equation (3) can be easily solved by the generalized eigenvalue decomposition approach as follows:

(4) $${\mathrm{MY}}^{T}{=\lambda Y}^{T}$$

Then, we can obtain the eigenvector corresponding to the dth smallest non-zero eigenvalues, and the low-dimensional embedding matrix can be represented as $Y=\left[y_{1},\cdots {,y}_{d}\right]$.

The LLE algorithm [22] can successfully maintain the local neighbor geometric structure and have a fast calculation speed. However, as the number of data dimension and data size increases, it has large sparsity, poor noise, and other problems.

### 2.2. Laplacian Eigenmaps

Given the data set $X=\left[x_{1},\cdots {,x}_{N}\right]\in R^{D\times N}$ and using the KNN method to find the *k*-nearest neighbors of the sample $x_{i}$, an overall data structure matrix is then formed, and $x_{j}$ is the jth nearest sample of $x_{i}$. Then, there is a weight value as $h_{\mathrm{ij}}=\exp\left(-\left\|x_{i}{-x}_{j}\right\|\right)$. Let $Y=\left[y_{1},\cdots {,y}_{N}\right]\in R^{d\times N}$, which denotes the low-dimensional embedding samples of data set *X*, and ${Y=P}^{T}X$. Then, *Y* can be solved by constructing the following optimization problem [23]:

(5) $$\left\{ \begin{array}{l} \min\sum_{i,j=1}^{N}{\left\|y_{i}{-y}_{j}\right\|}^{2}h_{\mathrm{ij}}\\ s.t\sum_{i=1}^{N}y_{i}=0,\sum_{i=1}^{N}{\left\|y_{i}\right\|}^{2}=I \end{array}\right.$$

Similarly, Equation (5) constraints ensure it has a solution. And it can be solved by using the generalized eigenvalue decomposition approach as follows:

(6) Ly=λDy

where $D_{\mathrm{ii}}=\sum_{j}h_{\mathrm{ij}}$ is a diagonal matrix and L=D−H is the Laplacian matrix. H is the weight matrix made up of $h_{\mathrm{ij}}$. The embedding samples in the *d*-dimensional space are constructed by the eigenvectors corresponding to the *d* minimum eigenvalues.

The LE algorithm [24] introduces the graph theory to achieve the purpose of DR methods. Nevertheless, due to the inaccurate weight matrix in the LE algorithm, the traditional LE algorithm cannot accurately describe the structure for complex hyperspectral data, resulting in the fact that the data in the low-dimensional space cannot fully express the original data features.

## 3. Improved Spatial–Spectral Weight Manifold Embedding

To solve large sparsity, inexact weight, and other problems of the LLE and LE [25] algorithms, an improved spatial–spectral weight manifold embedding (ISS-WME) algorithm is proposed in this paper. It combines spatial–spectral and high-dimensional manifold structure information to construct a weight matrix corresponding to the HSI structure. Considering the multi-manifold structure of HSI, the combination of its structure and the nearest neighbor samples simultaneously makes the data neighbor relationship invariable, without breaking the original structure when embedding to the low-dimensional space. In this regard, Section 3.1 specifically analyzes how to construct a weight matrix that is more consistent with the sample structure. In addition, Section 3.2 describes the final optimization objective function.

### 3.1. Spatial–Spectral Weight Setting

Through experimental study, researchers have found that classification accuracy can be improved by combining the spatial information in the analysis of HSI. Hence, the ISS-WME method is based on spatial and spectral information. It uses the variation of Gaussian function to represent the spatial and spectral distance, respectively. Given the HSI data set ${X=[x}^{f}{,x}^{p}]$, where $x^{f}$ is the spectral reflectance of a pixel and $x^{p}$ is the spatial coordinates of a pixel, to construct $D_{\mathrm{ij}}$, we find each pair of samples $x_{i}{=[x}_{i}^{f}{,x}_{i}^{p}]$ and $x_{j}=[x_{j}^{f},x_{j}^{p}]$, where i,j=1,⋯,N. Therefore, the spatial distance matrix and spectral distance matrix are represented, respectively, as follows:

(7) $$\left\{ \begin{array}{l} D_{\mathrm{ij}}^{f}=1-\exp\left(-\frac{{\left\|x_{i}^{f}{-x}_{j}^{f}\right\|}^{2}}{\sigma_{f}^{2}}\right)\\ D_{\mathrm{ij}}^{p}=1-\exp\left(-\frac{{\left\|x_{i}^{p}{-x}_{j}^{p}\right\|}^{2}}{\sigma_{p}^{2}}\right) \end{array}\right.$$

Therefore, the spatial–spectral distance weight matrix is as follows:

(8) $$D_{\mathrm{ij}}{=D}_{\mathrm{ij}}^{f}\cdot D_{\mathrm{ij}}^{p}$$

In HSI, adjacent pixels in the same homogenous region usually belong to the same class, so any sample in the same class can be linearly represented by homogeneous neighbor samples. Similarly, the whole data sample centers can be represented by different classes of sample centers [26]. Hence, the HSI still maintains this characteristic after DR. We want to obtain a within-class representation coefficient matrix by minimizing the error of the collaboration representation model. To prevent overfitting, regularization constraints are added to the optimization model. The objective function of the within-class collaboration representation model is as follows:

(9) $$\sum_{k=1}^{c}\sum_{\begin{array}{l} i=1\\ i\in \tau_{k} \end{array}}^{l_{k}}\left({\left\|P^{T}x_{i}-P^{T}X_{k}\theta_{k}^{w}\right\|}_{2}^{2}+\lambda {\left\|\theta_{k}^{w}-\bar{\theta_{k}^{w}}\right\|}_{2}^{2}\right)$$

In Equation (9), $l_{k}$ is the sample number in the kth class and $\tau_{k}$ is the sample set other than the kth class, and $X_{k}$ is expressed as the sample set from the same class as $x_{i}$, except $x_{i}$. $\theta_{k}^{w}$ is the within-class linear representation coefficient matrix of the kth class sample, and the within-class mean coefficient matrix is $\bar{\theta_{k}^{w}}=\left[\frac{1}{n-1},\cdots ,\frac{1}{n-1}\right]\in R^{\left(n-1\right)\times 1}$, and $\theta^{w}$ denotes all the within-class linear representation coefficients $\left[\theta_{k}^{w}|i=1\cdots c\right]$.

Likewise, the objective function of the between-class representation coefficient matrix is as follows:

(10) $$\sum_{k=1}^{c}\left({\left\|P^{T}\bar{x}-P^{T}{\bar{X}}_{k}\theta_{k}^{b}\right\|}_{2}^{2}+\lambda {\left\|\theta_{k}^{b}-\bar{\theta_{k}^{b}}\right\|}_{2}^{2}\right)$$

In Equation (10), $\bar{x}$ is the mean of the total samples, and ${\bar{X}}_{k}$ is the central sample set of each class sample. $\theta_{k}^{b}$ denotes the between-class representation coefficient matrix of the *k*th class sample, and the between-class mean coefficient matrix is $\bar{\theta_{k}^{b}}=\left[\frac{1}{n-1},\cdots ,\frac{1}{n-1}\right]\in R^{\left(n-1\right)\times 1}$, and $\theta^{b}$ denotes all the between-class representation coefficients $\left[\theta_{k}^{b}|k=1\cdots c\right]$.

The within-class representation matrix $\theta_{k}^{w}$ is obtained by solving the minimum value of Equation (9) (setting the derivative of objective function about within-class representation coefficients to be 0):

(11) $$\frac{\partial f}{\partial \theta_{k}^{w}}=-2Y_{k}^{T}\left(x_{i}-Y_{k}\theta_{k}^{w}\right)+2\lambda \left(\theta_{k}^{w}-\bar{\theta_{k}^{w}}\right)=0$$

Therefore, the within-class coefficient matrix is as follows:

(12) $$\theta_{k}^{w}={\left(Y_{k}^{T}Y_{k}+\lambda I\right)}^{-1}\left(Y_{k}^{T}y_{i}+\lambda \bar{\theta_{k}^{w}}\right)$$

In the same way, we can set the derivative of objective function about between-class representation coefficients to be 0, so the between-class coefficient matrix is as follows:

(13) $$\theta_{k}^{b}={\left({\bar{Y}}_{k}{}^{T}{\bar{Y}}_{k}+\lambda I\right)}^{-1}\left({\bar{Y}}_{k}{}^{T}{\bar{y}}_{k}+\lambda \bar{\theta_{k}^{b}}\right)$$

### 3.2. ISS-WME Model

Given the HSI data set $X=\left[x_{1},\cdots {,x}_{N}\right]{,x}_{i}\in R^{D}$, we assume that the projection matrix $P\in R^{D\times d}$ is expected to project the data X into the low-dimensional space. $Y=\left[y_{1},\cdots {,y}_{N}\right]{,y}_{i}\in R^{d}$ represents the samples in low-dimensional space, and ${Y=P}^{T}X$. As proposed by Wu [15], both of the distance and structural factors are taken into account in this paper. Then, we regard the spatial–spectral matrix as the distance weight $W_{ij}^{D}$ and coefficient matrices as the structure weight $W_{\mathrm{ij}}^{S}$, then $W_{\mathrm{ij}}^{D}$ and $W_{\mathrm{ij}}^{S}$ constitute the new weight matrix between samples, such as the following:

(14) $$\left\{ \begin{array}{l} W_{ij}=W_{ij}^{D}\cdot W_{ij}^{S}\\ W_{ij}^{D}=D_{ij}\\ W_{ij}^{S}=\beta \theta^{w}+\left(1-\beta \right)\theta^{b} \end{array}\right.$$

where β represents the proportion of the within-class matrix and the between-class matrix in the structure weight.

Furthermore, the high-dimensional data mapping to the low-dimensional space not only makes the local manifold structure unchanged, but also maintains the local neighbor relationship invariant. Introducing the weight of Equation (14) to increase the robustness of the model, the improved weight manifold embedding optimization problem is as follows:

(15) $$\left\{ \begin{array}{l} \min\frac{1}{2}\sum_{i,j=1}^{N}{\left\|y_{i}{-y}_{j}\right\|}^{2}W_{\mathrm{ij}}+\alpha \sum_{i=1}^{N}{\left\|y_{i}-\sum_{j=1}^{k}G_{\mathrm{ij}}y_{j}\right\|}^{2}\\ s.t\sum_{i=1}^{N}{\left\|y_{i}\right\|}^{2}=I,\sum_{i=1}^{N}y_{i}=0,\sum_{j=1}^{k}G_{\mathrm{ij}}=1 \end{array}\right.$$

where α is a compromise parameter. $W_{ij}$ is the spatial–spectral matrix in Equation (14) and $G_{ij}$ is still the weight matrix representing the nearest neighbor relationship. If $x_{i}$ and $x_{j}$ are neighbors, $G_{ij}=d_{G}(i,j)$ represents the geodesic distance; otherwise, $G_{ij}=0$. According to Equations (2) and (5), the optimization problem (15) is equivalent to the following:

(16) $$\begin{array}{l} \min\frac{1}{2}\sum_{i,j=1}^{N}{\left\|P^{T}x_{i}{-P}^{T}x_{j}\right\|}^{2}W_{\mathrm{ij}}+\alpha \sum_{i=1}^{N}{\left\|P^{T}x_{i}{-P}^{T}\sum_{j=1}^{k}G_{\mathrm{ij}}x_{j}\right\|}^{2}\\ =\sum_{i,j=1}^{N}P^{T}\left(x_{i}W_{\mathrm{ij}}x_{i}^{T}{-x}_{i}W_{\mathrm{ij}}x_{j}^{T}\right)P+\alpha \sum_{i=1}^{N}P^{T}{\left({\mathrm{XI}}_{i}{-\mathrm{XG}}_{i}\right)}^{2}P\\ =\mathrm{tr}\left(P^{T}X\left(D^{'}{-W}^{\prime }\right)X^{T}P\right)+\alpha \mathrm{tr}\left(P^{T}X{\left(I-G\right)}^{T}\left(I-G\right)X^{T}P\right)\\ =\mathrm{tr}\left(P^{T}{\mathrm{XL}}^{\prime }X^{T}P\right)+\alpha \mathrm{tr}\left(P^{T}{\mathrm{XM}}^{\prime }X^{T}P\right)\\ =\mathrm{tr}\left(P^{T}{\mathrm{XBX}}^{T}P\right) \end{array}$$

where $L^{\prime }{=D}^{\prime }{-W}^{\prime }$ is the Laplacian matrix, $D_{\mathrm{ii}}^{\prime }=\sum_{j=1}^{N}W_{\mathrm{ii}}$ is a diagonal matrix, $W^{\prime }={\left[W_{ij}\right]}_{N\times N}$ is a symmetric matrix, and $M^{\prime }={\left(I-G\right)}^{T}\left(I-G\right)$. Moreover, $B=L^{\prime }+\alpha M^{\prime }$. Finally, the objective function can be conducted as the following optimization problem:

(17) $$\left\{ \begin{array}{l} \min tr\left(P^{T}XBX^{T}P\right)\\ s.t.P^{T}XD^{\prime }X^{T}P=I \end{array}\right.$$

With the method of Lagrange multiplier, the optimization problem is formed as follows:

(18) $$XBX^{T}p_{d}=\lambda^{\prime }XD^{\prime }X^{T}p_{d}$$

where $p_{d}$ is the generalized eigenvector of Equation (18) according to their eigenvalue $\lambda_{1}^{\prime }\leq \cdots \leq \lambda_{d}^{\prime }$. Then, we can learn a projection matrix $P=\left[p_{1},\cdots {,p}_{d}\right]$. In summary, Algorithm 1 is as follows:
**Algorithm 1** Process of the ISS-WME AlgorithmInput: HSI data set $X=\left[x_{1},\cdots {,x}_{N}\right]\in R^{D\times N}$ and $x_{i}=\left(x_{i}^{f}{,x}_{i}^{p}\right)$, low-dimensional space d≪D, *K* is the nearest neighbor. 1: HSI is segmented into superpixels using the SLIC segmentation method and randomly select training samples (for Pavia University, training samples are 2%, 4%, 6%, 8%, 10%), and then use Equations (7) and (8) to calculate the spatial–spectral distance matrix between superpixels. In addition, make sure the number of superpixels and training samples is the same. 2: Then, use Equations (12) and (13) to obtain the structure representation matrix between training samples. The product of the two types of matrices is taken as the new matrix Equation (14). 3: According to the local manifold structure and nearest neighbor relationship of the samples, the objective function of Equation (16) is constructed. 4: By solving the generalized feature of Equation (18), the corresponding eigenvector is obtained. 5: Learn a projection matrix *P*. Output: The data in low-dimensional space is ${Y=P}^{T}X$


## 4. Experiments and Discussion

In order to verify the effectiveness of the proposed algorithm ISS-WME, we conducted experiments on three commonly used HSI data sets, namely Indian Pines, Pavia University, and Salinas scene. We considered the overall accuracy (OA) [19], classification accuracy (CA), average accuracy (AA), and kappa coefficient (kappa) [27] of the classification results as evaluation values. We compared the ISS-WME algorithm with six other representative DR algorithms, i.e., PCA, Isomap [28], LLE, LE, SSSE, and WLE-LLE. We used two more commonly used classifiers, i.e., the Euclidean distance-based k-nearest neighbor (KNN) algorithm [29] and the support vector machine (SVM), to classify the low-dimensional data. We performed the experiment using MATLAB on an Intel Core CPU 2.59 GHz and 8 GB RAM computer.

### 4.1. Data Sets and Parameter Setting

#### 4.1.1. Data Sets

The Indian Pines, Pavia University, and Salinas scene data sets were subjected to experiments in the paper.

The Indian Pines data set [30,31] and Salinas scene data set [2,30] were the scenes gathered by the Airborne Visible/Infrared Imaging Spectrometer (AVIRIS) sensor. Indian Pines consisted of 145×145 pixels and 220 spectral bands. However, several spectral bands with noise and water absorption phenomena were removed from the data set, leaving a total of 200 radiance channels to be used in the experiments. Salinas had 512×217 pixels and 204 spectral bands.

The Pavia University data set [30,32] was acquired by the Reflective Optics System Imaging Spectrometer (ROSIS) sensor. Its size was 610×340 pixels. Some channels were removed due to noise and the remaining number of spectral bands was 103.

#### 4.1.2. Experimental Parameter Settings

For this paper, six different DR algorithms were compared with the proposed ISS-WME method. These comparison algorithms are described as follows. PCA, Isomap, LLE, and LE are four classical DR algorithms. The SSSE algorithm combines the spectral and spatial information and WLE-LLE combines the spectral and structural information. In addition, for the LE, LLE, WLE-LLE, and SSSE algorithms, the number of nearest neighbor samples must be set in the experiment. To compare and analyze the classification results in the experiment, the nearest neighbor samples were set as 15 in all experiments. The SSSE and ISS-WME algorithms also require computational spatial and spectral information, so we set the parameters as $\left(\sigma_{f},\sigma_{p}\right)=\left(0.1,100\right)$.

In each experiment, each data set was divided into training samples and testing samples. We used different DR algorithms to learn a projection matrix on the training samples, and then utilized the acquired embedding matrix to project the testing samples into the low-dimensional space. Finally, we used a KNN or SVM classifier to classify the data in the low-dimensional space. Moreover, to reduce the systematic error, the results were computed 10 times to calculate the average value for each experimental result with the associated standard deviation. We used OA, CA, AA, and K to evaluate the different algorithm performances. In the Indian Pines experiment, the parameters were set to (β,α)=(0.5,0.2). In the same way, the parameters were set to (β,α)=(0.5,0.1) in the Pavia University experiment. Finally, in the Salinas scene, the parameters were (β,α)=(0.5,0.2).

### 4.2. Results for the Indian Pines Data Set

To fully attest the algorithm performance of the ISS-WME method, experiments were carried out under the conditions of different numbers of training samples, different embedding dimensions, and different DR methods. We randomly selected n% (n = 10, 20, 30, 40, 50) samples from each class as the training sample set, and the rest were the testing sample set. We also set the hyperspectral dimensionality (HD) of low-dimensional embedding from 10 to 50. The results of the proposed ISS-WME method were compared with those of the other comparison DR methods.

**Figure 1:**
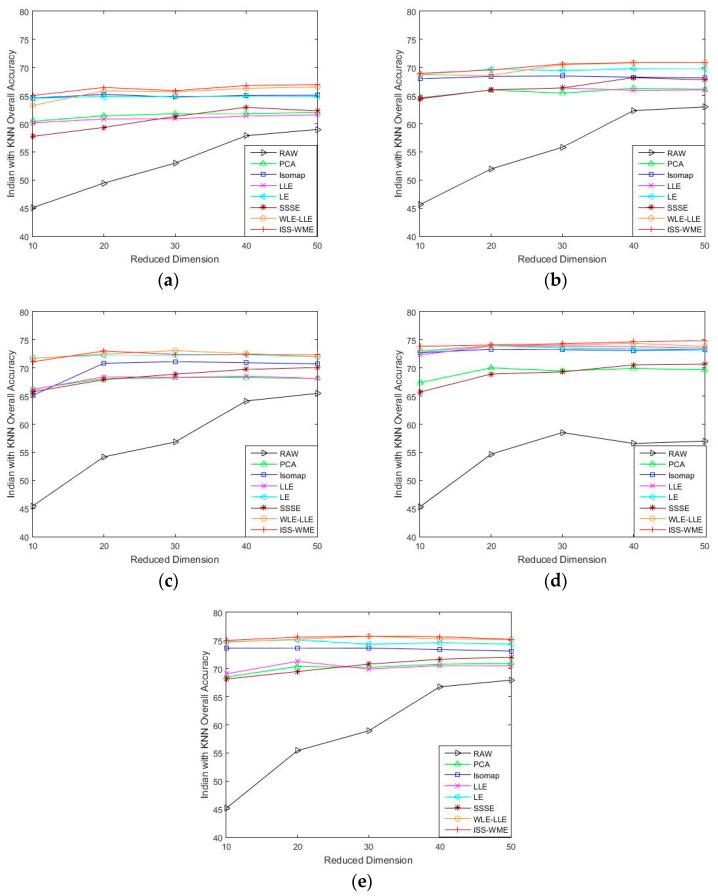
OA obtained by using a k-nearest neighbor (KNN) classifier, with respect to (**a**–**e**), different numbers of training sets (10%, 20%, 30%, 40%, 50%) and different dimensions (from 10 to 50) for the Indian Pines data set.

**Figure 2:**
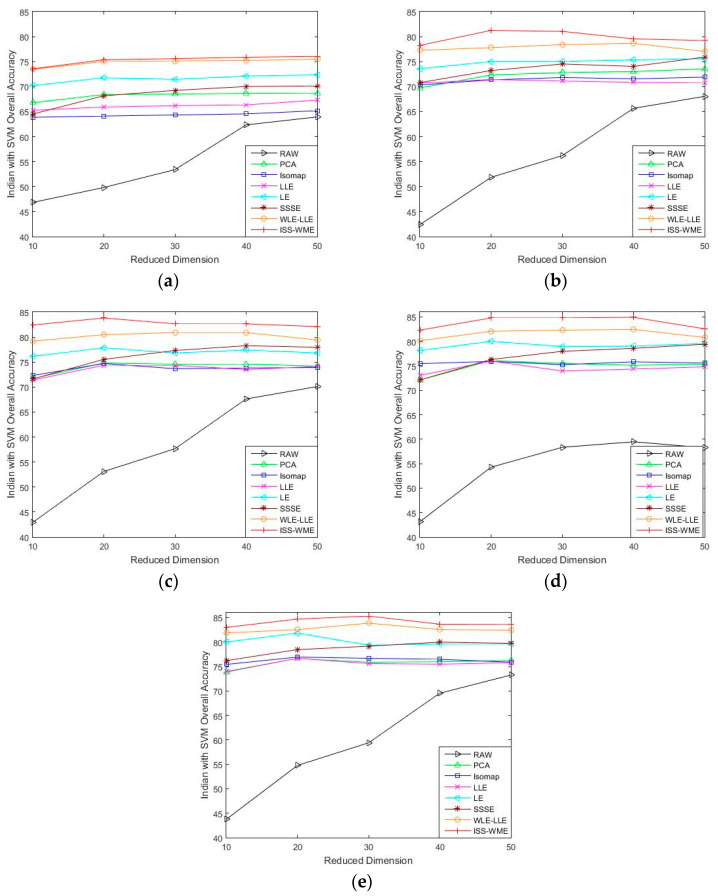
OA obtained by using a support vector machine (SVM) classifier, with respect to (**a**–**e**), different sizes of training sets (10%, 20%, 30%, 40%, 50%) and different hyperspectral dimensionality (HD) (from 10 to 50) for the Indian Pines data set.

Figure 1 and Figure 2 show the OA of the KNN and SVM classifiers on different embedding dimensions using different DR methods. Specifically, (a)–(e) represent different training sample sets. The OA of Indian Pines with different training samples directly classified by a KNN or SVM classifier was used as the baseline. Compared with the five other dimensionality reduction methods, ISS-WME and WLE-LLE achieved the best and the second-best overall accuracy, respectively, under different dimensions or different training samples. Comparing Figure 1 and Figure 2, the overall accuracy of SVM is higher than KNN.

**Figure 3:**
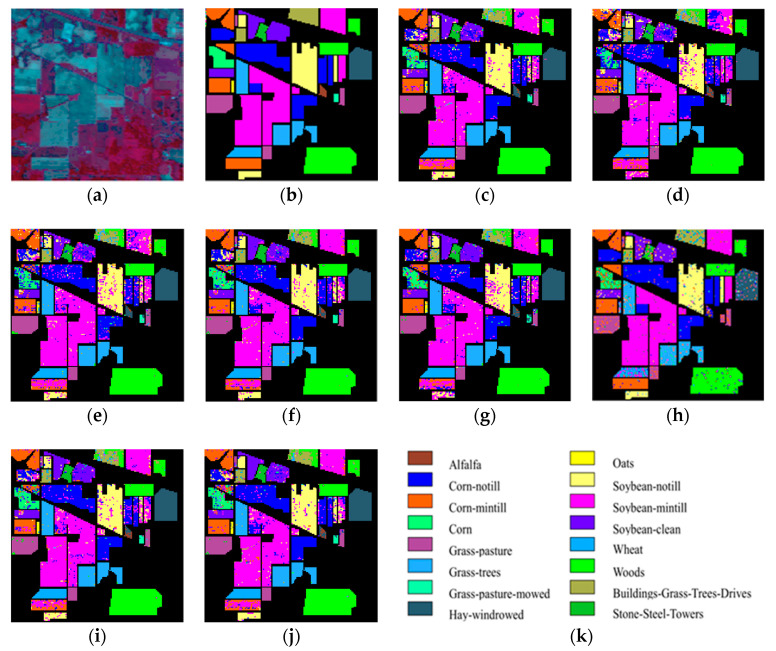
Classification maps of an SVM classifier using different dimensionality reduction (DR) algorithms for the Indian Pines data in HD = 20: (**a**) false-color image (R:57,G:27,B:17); (**b**) ground-truth map; (**c**) original (SVM); (**d**) principal component analysis (PCA); (**e**) Isomap; (**f**) local linear embedding (LLE); (**g**) Laplacian eigenmaps (LE); (**h**) SLIC superpixel based for Schroedinger eigenmaps (SSSE); (**i**) weighted local linear embedding (WLE-LLE); and (**j**) improved spatial–spectral weight manifold embedding (ISS-WME); (**k**) representation of different classes.

As can be seen in Figure 1 and Figure 2, the OA decreases as the dimension increases. In Figure 1, it can be observed that, for the KNN classifier, the proposed ISS-WME method obtains similar classification results with those of WLE-LLE in almost all cases of embedding dimensions, and achieves the best classification result in hyperspectral dimensionality (HD) = 50. Figure 2c shows the OA of the HD for the 30% samples of the Indian Pines data as training set. Compared with RAW, PCA, Isomap, LLE, LE, SSSE, and WLE-LLE, when HD = 50, ISS-WME increases the OA by 12.01%, 8.2%, 7.98%, 5.28%, 4.15%, and 2.69%, respectively. To further demonstrate intuitively the classification results of the DR algorithms, the comparison results for the 50% of the Indian Pines data trained with the SVM classifier in HD=20 are presented visually in Figure 3 with the best overall accuracy. It includes (a) the false-color image, (b) the corresponding ground-truth map, and the different DR methods’ classification maps (c)–(j). It can be observed that the proposed ISS-WME algorithm performs better in land-over classes than the other compared DR methods.

In order to further describe the comparison results, the quantitative comparison of classification accuracy using SVM classifiers under HD = 20 for different DR methods is summarized in Table 1. The results include the OA and kappa coefficient for each method, and each result is the average of the results of 10 runs with the associated standard deviation. As can be seen in Table 1, in most cases, the classification results (OA and kappa) generated by ISS-WME are the best.

Table 2 provides the training and testing sample numbers of each class in the Indian Pines data set in the experiment, as well as the classification results of the SVM classifier using different DR methods. Compared to Table 1, Table 2 shows the evaluation index CA, where the best results are shown in bold numbers. It can also be seen in Table 2 that the ISS-WME method achieves the best accuracy in 10 classes of samples.

### 4.3. Results for the Pavia University Data Set

In order to fully attest the algorithm performance of ISS-WME, experiments were carried out under the conditions of different numbers of training samples, different embedding dimensions, and different DR methods. We randomly selected *n*% (*n* = 2, 4, 6, 8, 10) samples from each class as the training set, and the rest were the testing set. We also set the hyperspectral dimensionality (HD) of low dimensional embedding from 10 to 50. The results of the proposed ISS-WME method were compared with those of the other DR methods.

**Figure 4:**
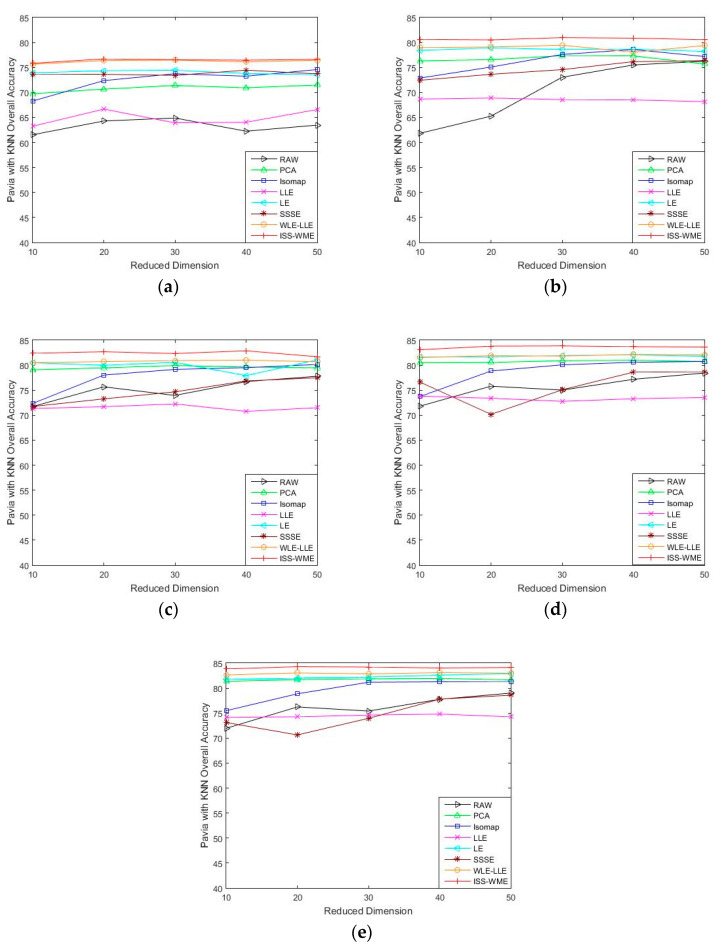
OA obtained by using a KNN classifier, with respect to (**a**–**e**), different numbers of training sets (2%, 4%, 6%, 8%, 10%) and different HD (from 10 to 50) for the Pavia University data set.

**Figure 5:**
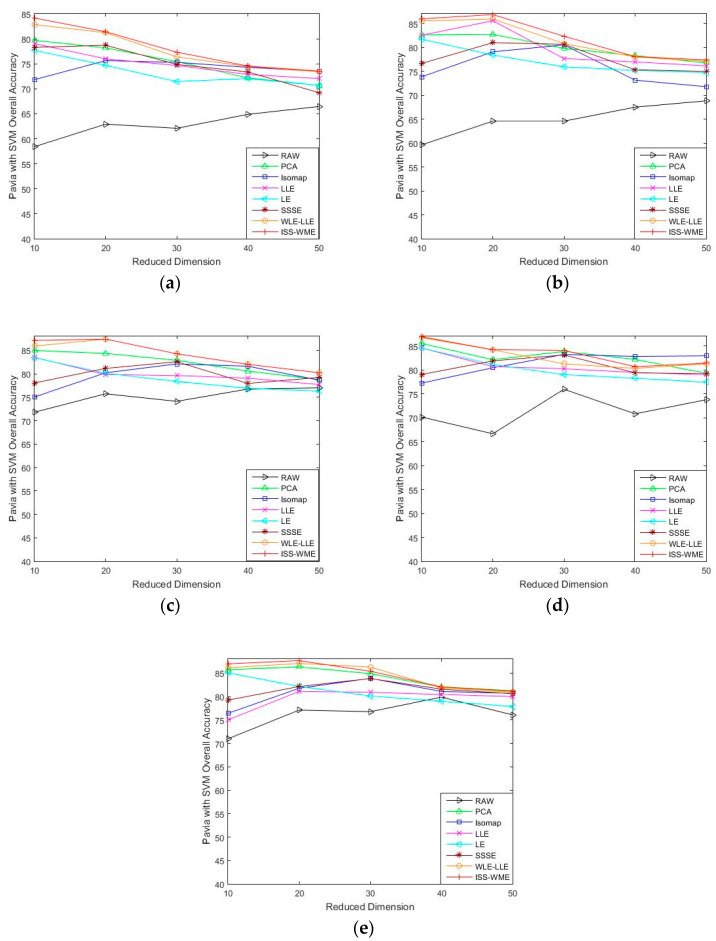
OA with respect to (**a**–**e**), different sizes of training sets (2%, 4%, 6%, 8%, 10%) and different HD (from 10 to 50) for the Pavia University data set, combined with the SVM classifier.

Figure 4 and Figure 5 show the OA of the KNN and SVM classifiers on different embedding dimensions using different DR methods. Specifically, (a)–(e) represent different training sets. The OA directly obtained by using different classifiers in dimensions was used as the baseline. Compared to the six other algorithms, ISS-WME achieved the best OA in almost all cases with different embedding dimensions under different numbers of training samples. As can be seen in Figure 5, image classification accuracies are more or less susceptible to distortion with the increase in embedding dimensions. No matter which DR algorithms are adopted, the curse of dimensionality occurs to a certain extent. Compared with Figure 4 and Figure 5, the distortion is serious when using the SVM classifier.

**Figure 6:**
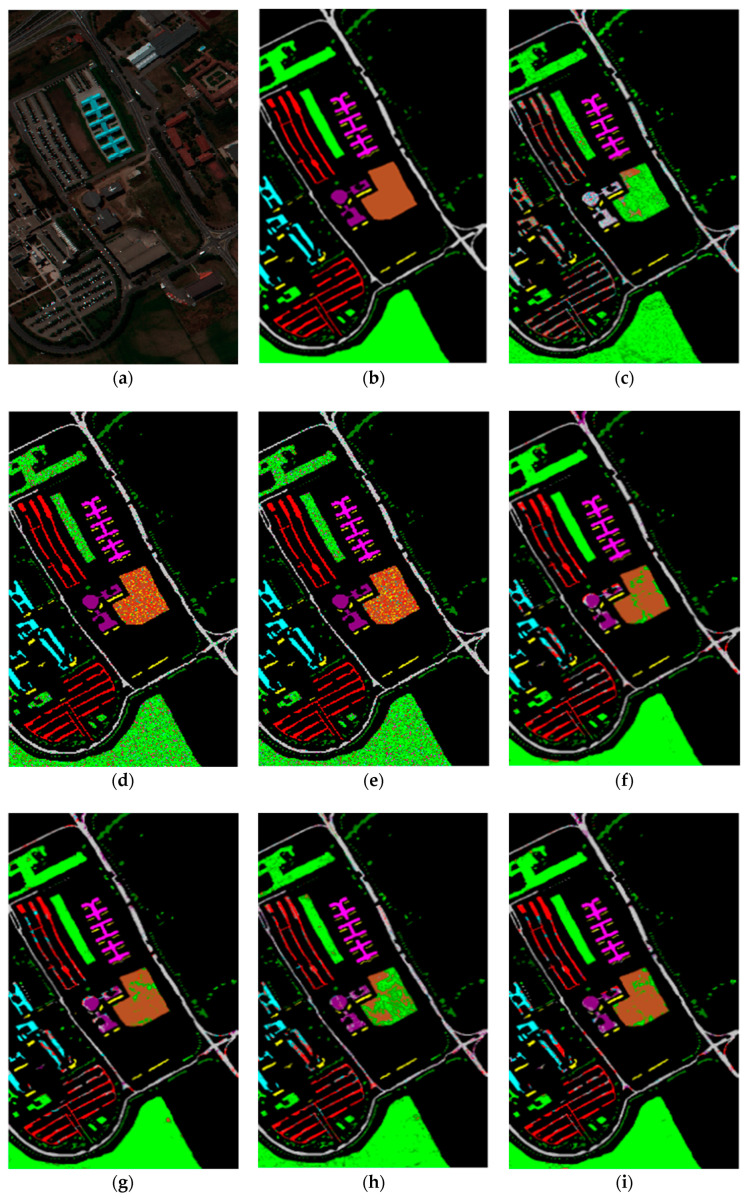

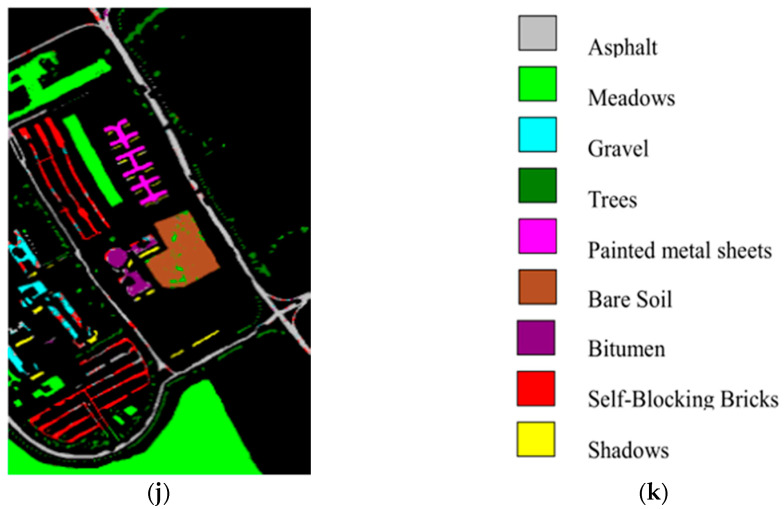
SVM classification maps of the different methods with the Pavia University data set in HD=20: (**a**) false-color image (R:102,G:56,B:31), (**b**) ground-truth map, (**c**) original (SVM), (**d**) PCA, (**e**) Isomap, (**f**) LLE, (**g**) LE, (**h**) SSSE, (**i**) WLE-LLE, and (**j**) ISS-WME; (**k**) representation of different classes.

As can be seen in Figure 4, the OA of different DR methods is relatively stable with the increase in training sets when KNN is used as the classifier. Figure 5e shows the impact of the hyperspectral dimensionality (HD) on the OA for 10% samples of Pavia University data as training set. Compared with RAW, PCA, Isomap, LLE, LE, SSSE, and WLE-LLE, when HD = 50, ISS-WME increases the OA by 0.13%, 0.55%, 1.24%, 3.34%, 0.64%, and 0.31%, respectively. In order to further demonstrate the classification results of DR algorithms, the classification result maps for the 10% of the Pavia University data trained with the SVM classifier in HD = 20 are presented visually in Figure 6, including (a) the false-color image, (b) the corresponding ground-truth map, and the different DR methods’ classification maps (c)–(j). It can be observed that the proposed ISS-WME algorithm performs better than the other compared DR methods, in most land-over classes.

To further describe the comparison results, the quantitative comparison of OA of different DR methods at HD = 20 is summarized in Table 3. The results include the overall accuracy and kappa coefficients of each method, and each result is an average of the results of 10 runs with the associated standard deviation. As can be seen in Table 3, the classification results (OA and kappa) produced by ISS-WME are the best in most cases. In addition, it can be seen in Table 4 that ISS-WME obtained the best classification accuracy about six classes, and the best results of the indexes are shown in bold.

Table 4 provides the number of training and test samples for each class in the Pavia University data set in the experiment, as well as the classification results under the SVM classifier using different dimensionality reduction methods. Compared with Table 3, the classification accuracy (CA) is displayed in Table 4, where the best results are shown in bold numbers. Moreover, it can be seen in Table 4 that the ISS-WME method achieves the best accuracy in sixclasses of samples.

### 4.4. Results for the Salinas Scene Data Set

To describe the comparison results, the quantitative comparison of OA of different DR methods when HD = 20 is summarized in Table 5. The results include the overall accuracy and kappa coefficients of each method, and each result is an average of the results of 10 runs with the associated standard deviation. As can be seen in Table 5, the classification results (OA and kappa) produced by ISS-WME are the best in most cases. In addition, it can be seen in Table 6 that ISS-WME obtained the best classification accuracy about 12 classes, and the best results of the indexes are shown in bold. Moreover, the results of three classes are the same as the WLE-LLE algorithm.

Table 5 provides the number of training and test samples for each class in the Salinas scene data set in the experiment, as well as the classification results under the SVM classifier using different dimensionality reduction methods. Compared with Table 5, the classification accuracy (CA) is displayed in Table 6, where the best results are shown in bold numbers. And the visual representation of different dimensional reduction methods of Salinas data set is supplemented in the Appendix A.

## 5. Conclusions

In this paper, a dimensionality reduction method combining the manifold structure of high-dimensional data with a linear nearest neighbor relationship was proposed. The method aimed to keep the data nearest neighbor relationship unchanged when the high-dimensional data were projecting to the low-dimensional space. Furthermore, the manifold structure of the data combined the spatial–spectral distance and structural features. To fully verify the superiority of the proposed method, the data obtained by the ISS-WME method and the six other dimensionality reduction methods were classified by two common classifiers. The results of several experiments show that the ISS-WME algorithm improves the ground object recognition ability of hyperspectral data, and the OA and kappa coefficients also support this conclusion. In the future, the dimensionality reduction labeling will be further considered to improve the classification effect through the framework of semi-supervised learning.

## Figures and Tables

**Table 1:** Results of the different DR methods for the Indian Pines data set (OA%±ASD%).

Samples	Classifier	Index	RAW	PCA	Isomap	LLE	LE	SSSE	WLE-LLE	ISS-WME
10%	KNN	OA	49.44 ± 1.94	61.42 ± 1.35	65.23 ± 1.62	60.85 ± 1.30	64.82 ± 1.47	59.35 ± 1.96	65.86 ± 1.39	66.46 ± 1.90
		Kappa	32.79 ± 1.65	44.98 ± 1.15	49.26 ± 1.59	44.21 ± 1.59	49.42 ± 1.89	42.95 ± 2.11	48.31 ± 1.56	48.86 ± 1.85
	SVM	OA	49.82 ± 1.37	68.40 ± 1.14	64.12 ± 1.62	65.93 ± 1.71	71.77 ± 1.61	68.17 ± 1.10	75.03 ± 1.26	75.38 ± 1.47
		Kappa	33.19 ± 1.35	52.82 ± 1.11	48.26 ± 1.65	51.94 ± 1.23	56.19 ± 1.51	52.93 ± 1.03	59.71 ± 1.35	62.08 ± 1.56
20%	KNN	OA	51.97 ± 1.17	66.00 ± 1.48	68.42 ± 1.35	66.00 ± 1.20	69.72 ± 1.35	66.03 ± 1.74	68.60 ± 1.43	69.56 ± 1.71
		Kappa	32.56 ± 1.52	50.10 ± 1.59	52.90 ± 1.25	50.20 ± 1.23	54.37 ± 1.36	50.44 ± 1.71	53.42 ± 1.41	54.27 ± 1.75
	SVM	OA	51.86 ± 1.59	72.34 ± 1.47	71.36 ± 1.62	71.42 ± 1.27	75.01 ± 1.75	73.22 ± 1.45	77.80 ± 1.93	81.25 ± 1.51
		Kappa	35.08 ± 1.65	57.15 ± 1.42	55.74 ± 1.79	56.15 ± 1.39	58.56 ± 1.67	58.13 ± 1.53	62.83 ± 1.92	66.29 ± 1.42
30%	KNN	OA	54.19 ± 1.28	68.13 ± 1.64	70.83 ± 1.53	68.43 ± 1.66	72.30 ± 1.32	67.91 ± 1.03	72.46 ± 1.35	73.02 ± 1.43
		Kappa	37.55 ± 1.36	52.60 ± 1.74	55.54 ± 1.56	52.94 ± 1.51	57.29 ± 1.22	52.48 ± 1.87	57.34 ± 1.18	57.84 ± 1.55
	SVM	OA	53.11 ± 1.35	74.82 ± 1.69	74.72 ± 1.33	74.37 ± 1.48	77.83 ± 1.54	77.54 ± 1.33	80.48 ± 1.76	83.83 ± 1.73
		Kappa	36.51 ± 1.63	59.63 ± 1.66	59.45 ± 1.34	59.07 ± 1.56	63.60 ± 1.55	60.53 ± 1.19	65.57 ± 1.71	69.73 ± 1.71
40%	KNN	OA	54.67 ± 1.62	70.03 ± 1.13	73.33 ± 1.84	75.91 ± 1.47	73.94 ± 1.20	68.92 ± 1.14	73.94 ± 1.79	74.08 ± 1.44
		Kappa	38.36 ± 1.14	54.64 ± 1.10	58.26 ± 1.79	60.86 ± 1.43	59.05 ± 1.15	53.58 ± 1.35	58.99 ± 1.77	59.25 ± 1.50
	SVM	OA	54.28 ± 1.81	76.07 ± 1.44	75.91 ± 1.37	75.98 ± 1.21	80.02 ± 1.65	76.31 ± 0.96	82.07 ± 1.53	84.80 ± 1.80
		Kappa	37.72 ± 1.71	61.13 ± 1.46	60.94 ± 1.42	60.86 ± 1.23	63.00 ± 1.63	61.45 ± 0.93	67.35 ± 1.57	70.36 ± 2.34
50%	KNN	OA	55.41 ± 1.50	70.38 ± 1.44	73.67 ± 1.29	71.29 ± 1.47	75.10 ± 1.56	69.48 ± 1.51	75.22 ± 1.47	74.56 ± 1.36
		Kappa	39.14 ± 1.44	55.19 ± 1.40	58.71 ± 1.24	55.93 ± 1.41	60.31 ± 1.44	54.28 ± 1.50	60.27 ± 1.48	59.73 ± 1.24
	SVM	OA	54.77 ± 1.39	76.65 ± 1.76	76.93 ± 1.38	76.67 ± 1.27	81.84 ± 1.24	78.46 ± 1.59	82.54 ± 1.27	84.71 ± 0.93
		Kappa	38.12 ± 1.35	61.75 ± 1.76	61.89 ± 1.49	61.63 ± 1.34	66.89 ± 1.21	63.51 ± 1.77	67.74 ± 1.37	70.11 ± 1.06

**Table 2:** Results of each class of samples in different DR methods for the Indian Pines data set (HD = 20).

Class	Sample		DR + SVM Classifier (%)							
	Train	Test	RAW	PCA	Isomap	LLE	LE	SSSE	WLE-LLE	ISS-WME
Alfalfa	23	23	13.04	52.17	26.09	30.77	40.58	52.17	56.52	**86.96**
Corn-N	714	714	38.42	69.37	69.42	40.06	70.07	65.92	**77.08**	72.17
Corn-M	415	415	25.06	48.76	49.96	44.34	**62.33**	58.47	62.25	56.47
Corn	119	118	14.41	**77.11**	38.14	26.27	47.74	46.05	62.15	53.95
Grass-P	242	241	59.06	90.87	89.21	62.38	89.76	89.35	94.47	**94.65**
Grass-T	365	365	86.48	97.81	97.63	97.90	97.44	97.63	98.26	**98.86**
Grass-P-M	14	14	35.71	76.19	52.38	50.28	90.48	76.19	83.33	**83.81**
Hay-W	239	239	88.70	99.86	99.72	97.13	99.44	98.61	**99.72**	99.68
Oats	10	10	11.24	43.33	60.00	30.00	80.00	80.00	70.00	**86.67**
Soybean-N	486	486	25.17	63.51	69.82	**94.24**	78.26	79.08	75.03	75.17
Soybean-M	1228	1227	71.15	83.32	81.83	73.62	**87.48**	83.46	86.66	79.52
Soybean-C	297	296	56.41	64.75	57.32	52.70	67.91	60.47	73.99	**74.07**
Wheat	103	102	74.51	94.12	98.69	75.21	97.06	97.39	99.67	**99.87**
Woods	633	632	94.57	97.68	97.63	86.71	97.31	96.78	97.42	**97.66**
Buildings-G-T-D Stone-S-T	193	193	29.02	45.77	45.60	34.20	43.52	44.39	51.81	**52.85**
	47	46	91.30	90.58	94.20	88.70	86.96	92.03	96.38	**98.41**
OA			54.77	76.65	76.93	76.67	81.84	78.46	82.54	84.71
AA			54.04	74.70	70.47	61.53	77.27	76.12	80.30	81.92
kappa			38.12	61.75	61.89	61.63	66.89	63.51	67.74	70.11

**Table 3:** Results of the different DR methods for the Pavia University data set.

Samples	Classifier	Index	RAW	PCA	Isomap	LLE	LE	SSSE	WLE-LLE	ISS-WME
2%	KNN	OA	61.55 ± 1.64	69.75 ± 0.90	68.29 ± 2.20	63.25 ± 1.81	73.92 ± 1.27	73.70 ± 1.10	75.65 ± 1.47	75.84 ± 1.27
		Kappa	44.23 ± 1.23	57.78 ± 1.49	55.49 ± 3.13	47.71 ± 2.94	63.99 ± 1.17	63.31 ± 1.52	66.12 ± 1.29	66.38 ± 1.42
	SVM	OA	58.42 ± 1.13	79.71 ± 1.18	71.84 ± 3.22	79.05 ± 1.42	77.70 ± 1.82	78.30 ± 0.88	82.86 ± 0.83	84.17 ± 0.87
		Kappa	44.61 ± 2.96	71.99 ± 1.60	60.66 ± 4.61	70.84 ± 1.71	69.24 ± 2.66	69.91 ± 1.23	76.65 ± 1.13	78.32 ± 1.19
4%	KNN	OA	61.83 ± 1.46	76.32 ± 1.23	72.89 ± 1.76	68.69 ± 1.76	78.44 ± 1.70	72.45 ± 1.33	78.99 ± 1.26	80.64 ± 1.51
		Kappa	45.04 ± 1.06	67.18 ± 1.48	62.40 ± 1.25	56.39 ± 1.45	70.09 ± 1.84	61.89 ± 1.27	70.09 ± 1.45	73.34 ± 1.59
	SVM	OA	59.62 ± 1.51	82.60 ± 1.74	73.84 ± 3.43	82.52 ± 1.04	81.71 ± 1.21	76.63 ± 1.28	85.53 ± 1.16	85.96 ± 0.99
		Kappa	42.32 ± 1.19	76.07 ± 2.51	63.60 ± 5.03	76.12 ± 1.44	74.96 ± 1.25	67.80 ± 1.74	80.34 ± 1.29	80.95 ± 1.16
6%	KNN	OA	71.73 ± 1.39	79.08 ± 1.18	72.35 ± 1.83	71.35 ± 1.37	80.48 ± 1.29	71.74 ± 2.39	80.51 ± 1.50	82.38 ± 1.44
		Kappa	63.93 ± 1.80	71.16 ± 1.63	61.88 ± 1.96	59.82 ± 2.44	73.03 ± 1.42	60.56 ± 3.58	73.18 ± 1.58	75.76 ± 1.64
	SVM	OA	71.81 ± 1.27	84.98 ± 1.84	75.06 ± 1.86	85.32 ± 1.32	83.45 ± 1.46	78.05 ± 2.06	85.93 ± 1.69	87.13 ± 1.49
		Kappa	65.34 ± 1.61	79.49 ± 1.20	65.34 ± 2.78	77.38 ± 1.49	77.52 ± 1.71	69.81 ± 2.94	80.92 ± 1.93	82.56 ± 1.68
8%	KNN	OA	71.80 ± 1.51	80.45 ± 1.36	73.72 ± 1.89	73.81 ± 1.23	81.65 ± 1.16	76.63 ± 2.25	81.54 ± 1.51	83.09 ± 1.19
		Kappa	65.14 ± 1.83	73.11 ± 1.54	63.93 ± 2.61	63.96 ± 1.38	74.74 ± 1.23	62.39 ± 2.55	74.47 ± 1.74	76.79 ± 1.28
	SVM	OA	70.14 ± 1.22	85.52 ± 0.87	77.23 ± 2.55	84.56 ± 1.21	84.55 ± 1.30	79.03 ± 1.43	86.75 ± 1.30	86.91 ± 1.40
		Kappa	61.92 ± 1.46	80.31 ± 1.23	68.58 ± 3.72	78.96 ± 1.29	79.06 ± 1.42	71.17 ± 1.64	82.08 ± 1.43	80.20 ± 1.55
10%	KNN	OA	71.96 ± 1.18	81.36 ± 1.47	75.48 ± 1.56	74.15 ± 0.95	81.74 ± 0.72	73.11 ± 1.73	82.62 ± 1.46	83.83 ± 0.59
		Kappa	65.60 ± 1.46	74.21 ± 1.00	66.23 ± 2.24	64.20 ± 1.43	74.85 ± 1.02	62.48 ± 2.84	76.11 ± 1.69	77.85 ± 0.86
	SVM	OA	70.99 ± 1.31	85.75 ± 1.29	76.42 ± 0.65	75.02 ± 0.73	85.07 ± 1.18	79.24 ± 1.00	86.15 ± 0.24	86.98 ± 1.12
		Kappa	63.95 ± 1.29	80.68 ± 1.77	67.42 ± 1.19	59.69 ± 0.86	79.80 ± 1.31	71.46 ± 1.40	82.65 ± 0.93	82.37 ± 1.12

**Table 4:** Results of each class of samples in different DR methods for the Pavia University data set (HD = 20).

Class	Sample		DR+SVM Classifier (%)							
	Train	Test	RAW	PCA	Isomap	LLE	LE	SSSE	WLE-LLE	ISS-WME
Asphalt	657	6565	62.96	88.41	74.41	86.10	**95.19**	81.98	84.93	88.03
Meadows	1846	18463	91.90	97.72	95.76	96.66	**98.78**	96.19	96.50	97.42
Gravel	208	2078	45.72	50.71	47.89	59.04	80.93	52.38	64.55	**84.23**
Trees	303	3033	39.96	84.75	79.55	86.34	87.00	75.25	**89.90**	89.65
Metal sheets	133	1332	98.51	99.61	99.94	99.72	**100.00**	99.23	**100.00**	**100.00**
Bare Soil	498	4979	46.54	66.36	80.77	48.25	87.39	58.26	65.56	**87.44**
Bitumen	132	1317	45.02	49.72	42.47	65.27	79.88	66.16	74.02	**80.38**
Bricks	365	3645	54.43	84.45	75.44	83.27	84.25	80.57	82.78	**86.38**
Shadows	94	937	46.57	99.37	62.50	99.68	95.88	99.84	93.38	**100.00**
OA			70.99	85.75	76.42	75.02	85.07	79.24	86.15	86.98
AA			59.07	80.12	73.19	80.48	89.92	78.87	83.51	90.39
kappa			63.95	80.68	67.42	59.69	79.80	71.46	82.65	82.37

**Table 5:** Results of the different DR methods for the Salinas scene data set (HD = 20).

Samples	Classifier	Index	RAW	PCA	Isomap	LLE	LE	SSSE	WLE-LLE	ISS-WME
2%	KNN	OA	75.23 ± 1.64	75.98 ± 2.90	76.52 ± 2.20	82.56 ± 2.81	78.56 ± 1.27	80.12 ± 1.10	81.53 ± 1.47	82.67 ± 1.43
		Kappa	59.89 ± 1.23	65.12 ± 1.49	63.29 ± 3.13	72.69 ± 2.94	65.23 ± 1.17	69.96 ± 1.22	70.69 ± 2.29	69.12 ± 1.96
	SVM	OA	63.34 ± 1.13	75.12 ± 2.18	78.22 ± 3.22	89.23 ± 2.42	86.23 ± 1.72	86.23 ± 1.88	88.93 ± 0.83	88.53 ± 1.23
		Kappa	50.96 ± 2.96	64.36 ± 1.60	69.35 ± 4.61	79.96 ± 1.71	75.69 ± 2.66	74.15 ± 1.03	80.63 ± 1.53	75.63 ± 1.56
4%	KNN	OA	78.20 ± 1.86	76.52 ± 1.23	75.89 ± 1.66	83.63 ± 1.76	79.23 ± 1.70	81.23 ± 1.33	83.56 ± 1.16	83.84 ± 1.51
		Kappa	60.93 ± 2.06	64.78 ± 1.48	63.25 ± 1.85	72.56 ± 1.45	66.36 ± 1.84	70.36 ± 1.27	72.12 ± 2.05	70.34 ± 1.09
	SVM	OA	66.47 ± 1.51	77.25 ± 1.54	79.94 ± 2.43	89.38 ± 2.94	88.23 ± 1.71	89.23 ± 1.88	88.99 ± 1.06	90.22 ± 0.99
		Kappa	55.63 ± 2.19	65.23 ± 1.51	67.89 ± 4.03	78.96 ± 3.44	75.63 ± 1.85	75.63 ± 1.74	80.34 ± 1.29	80.95 ± 1.16
6%	KNN	OA	78.91 ± 1.39	76.23 ± 1.18	78.59 ± 1.83	85.26 ± 2.37	80.23 ± 2.29	83.23 ± 2.39	85.13 ± 1.23	85.02 ± 1.64
		Kappa	62.36 ± 2.80	63.63 ± 2.63	62.56 ± 1.96	74.23 ± 2.44	69.36 ± 2.42	70.32 ± 3.28	73.25 ± 1.78	72.76 ± 1.64
	SVM	OA	68.01 ± 1.27	77.56 ± 1.84	80.49 ± 1.86	91.61 ± 2.32	89.56 ± 1.86	90.12 ± 2.26	89.96 ± 1.29	91.90 ± 1.29
		Kappa	59.13 ± 1.61	68.96 ± 1.20	72.06 ± 1.78	80.65 ± 2.49	77.96 ± 1.71	76.12 ± 2.48	78.92 ± 1.63	82.56 ± 1.68
8%	KNN	OA	78.82 ± 1.51	76.63 ± 1.36	81.56 ± 1.89	85.96 ± 1.23	81.63 ± 2.16	84.63 ± 2.25	85.17 ± 1.31	86.23 ± 1.19
		Kappa	65.34 ± 1.83	63.59 ± 1.54	65.75 ± 2.61	75.26 ± 1.38	70.23 ± 1.23	72.12 ± 2.55	73.69 ± 1.54	73.79 ± 1.28
	SVM	OA	68.96 ± 2.22	77.96 ± 1.87	81.20 ± 2.55	91.92 ± 3.21	90.05 ± 1.30	88.96 ± 1.73	89.69 ± 1.30	91.16 ± 1.04
		Kappa	57.69 ± 2.46	65.36 ± 2.23	73.96 ± 3.72	79.86 ± 3.29	81.02 ± 1.42	78.02 ± 1.64	76.08 ± 1.23	80.20 ± 1.55
10%	KNN	OA	80.21 ± 1.18	77.69 ± 1.47	84.72 ± 1.56	86.95 ± 0.95	81.23 ± 1.72	85.23 ± 1.73	86.33 ± 1.46	86.78 ± 1.72
		Kappa	68.23 ± 1.46	65.26 ± 2.00	69.89 ± 2.24	67.36 ± 1.43	71.53 ± 2.02	72.36 ± 2.84	76.11 ± 1.69	72.19 ± 1.02
	SVM	OA	69.13 ± 1.21	79.02 ± 2.29	80.99 ± 0.65	92.16 ± 2.73	89.92 ± 2.18	90.13 ± 1.20	90.57 ± 1.24	92.19 ± 1.02
		Kappa	58.63 ± 1.09	68.32 ± 2.77	79.63 ± 2.19	81.96 ± 2.86	78.69 ± 1.91	76.98 ± 1.40	82.65 ± 1.93	84.23 ± 1.62

**Table 6:** Results of each class of samples in different DR methods for the Salinas scene data set (HD = 20).

Class	Sample		DR+SVM Classifier (%)							
	Train	Test	RAW	PCA	Isomap	LLE	LE	SSSE	WLE-LLE	ISS-WME
Brocoil_green_weeds_1	201	1808	91.26	93.14	99.34	96.68	99.23	98.23	**99.56**	98.01
Brocoil_green_weeds_2	373	3353	99.22	99.28	99.88	95.53	99.64	91.65	**99.88**	**99.88**
Fallow	198	1778	61.75	81.33	94.60	93.59	99.52	96.29	**99.78**	93.36
Fallow_rough_plow	139	1255	96.49	97.29	97.63	95.37	98.34	97.61	**99.36**	99.20
Fallow_smooth	268	2410	80.00	83.24	97.42	83.65	99.88	89.46	**98.34**	**98.34**
Stubble	396	3563	95.29	96.07	94.50	86.59	99.94	94.39	**100.00**	99.94
Celery	358	3221	97.21	89.67	90.18	88.33	88.62	98.63	99.44	**99.75**
Grapes_untrained	113	11158	75.75	83.28	88.86	83.59	97.82	87.38	84.27	**99.48**
Soil_vinyard_develop	620	5583	98.64	90.69	99.25	97.13	94.58	94.75	99.86	**99.89**
Corn_senesced_green_weed	328	2950	83.12	84.61	93.42	95.25	96.20	98.31	**99.17**	**99.17**
Lettuce_romaine_4wk	107	961	10.19	81.50	97.30	83.58	92.77	89.81	91.89	**99.77**
Lettuce_romaine_5wk	193	1734	90.26	92.17	99.88	91.47	98.79	94.23	96.77	**99.77**
Lettuce_romaine_6wk	92	824	94.90	97.57	98.06	92.73	90.23	97.09	98.06	**98.79**
Lettuce_romaine_7wk	107	963	78.17	99.38	97.77	89.81	58.45	94.39	92.52	**92.72**
Vinyard_untrained	727	6541	41.36	54.32	64.72	56.23	58.45	66.73	57.11	**67.25**
Vinyard_vertical_trellis	181	1626	87.71	98.40	98.65	98.53	97.26	97.54	96.65	**98.77**
OA			69.13	79.02	80.99	92.16	89.92	90.13	90.57	92.19
AA			80.08	88.87	94.47	89.25	91.86	92.91	94.54	96.51
kappa			58.63	68.32	79.63	81.96	78.69	76.98	82.65	84.23

**Figure A1:**
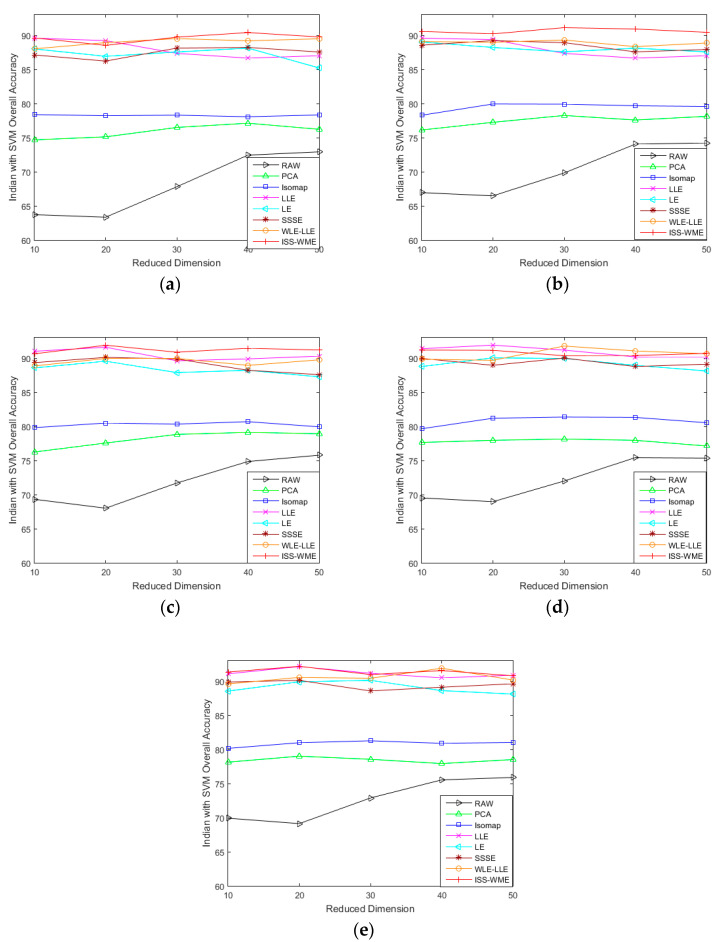
OA obtained by using an SVM classifier, with respect to (**a**–**e**), different numbers of training sets (2%, 4%, 6%, 8%, 10%) and different HD (from 10 to 50) for the Salinas scene data set.

**Figure A2:**
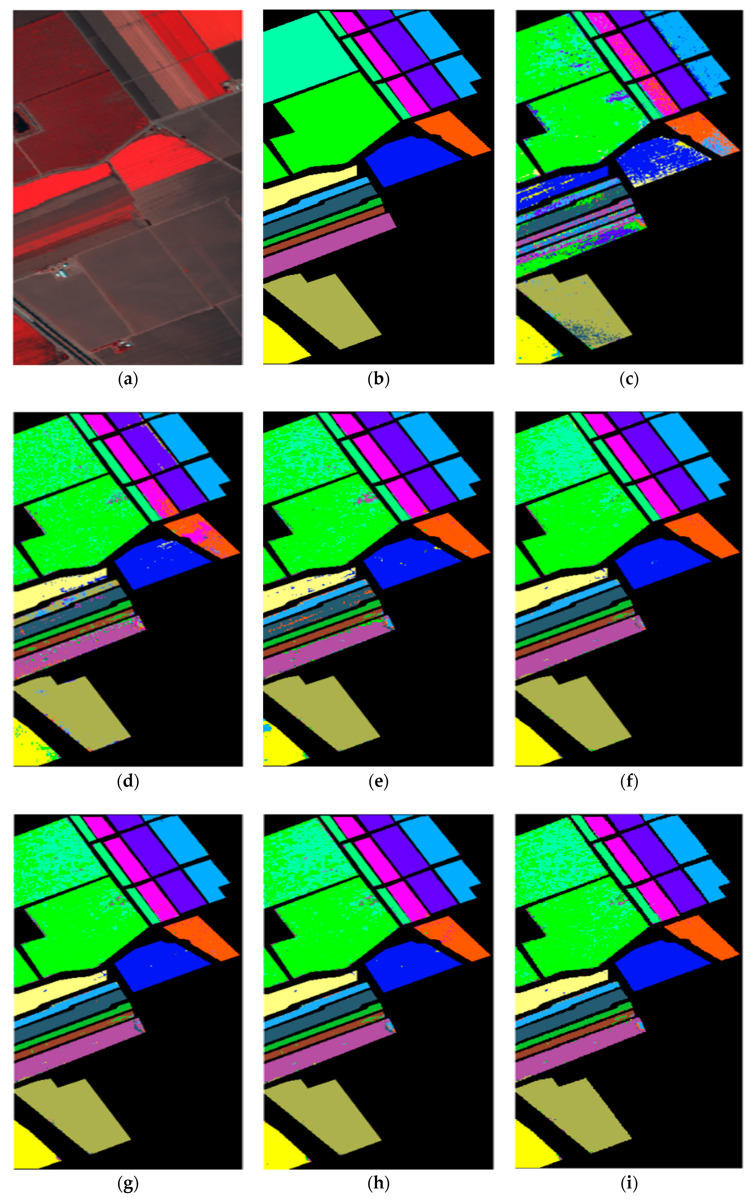

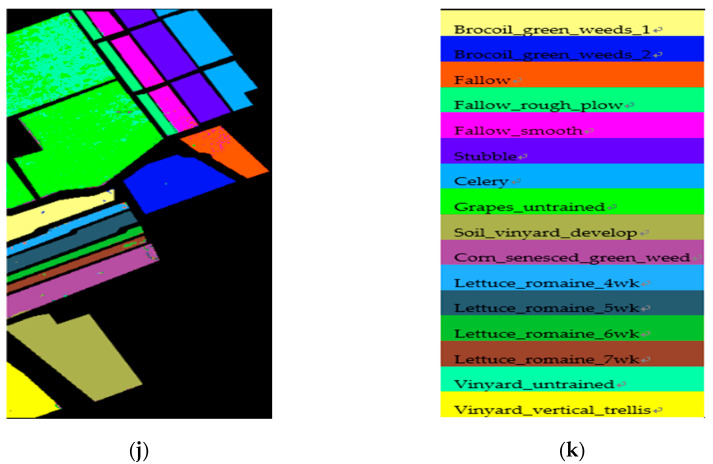
Classification maps of the different DR methods using an SVM classifier for the Salinas scene data in HD = 20: (**a**) false-color image (R:42,G:16,B:11), (**b**) ground-truth map, (**c**) original (SVM), (**d**) PCA, (**e**) Isomap, (**f**) LLE, (**g**) LE, (**h**) SSSE, (**i**) WLE-LLE, and (**j**) ISS-WME; (**k**) representation of different classes.
